# Event and Comment

**Published:** 1910-01-15

**Authors:** 


### DENTAL REGISTER.

**Vol. LXIV. January 15, 1910. No. 1.**

### EVENT AND COMMENT.

**SOME COMMENTS ON THE COUNTRY DENTISTS PROBLEM.** We made an editorial comment in the November *Register* on an article which appeared in the *Dental Digest* written by a dentist whose practice in a small country town was not sufficiently remunerative to enable him to save any of his earnings. The *December Digest* contains a number of letters on the same article and we have culled some of these and reprint here in abstract.

One writer suggests that there may be some profitable patients in the town who do not patronize the office, and recommends that a discreet office girl could be used to advantage in exerting an influence that will direct their attention to his place of business. He also suggests that it is bad business policy to live and have the office in the same "Flat," and that he should refurnish the office and let the people know it is being done; should wear better clothes and make an appearance of prosperity; should not begin office hours until 9 A. M., and quit at 4 P. M. Should have some leisure time for "mixing" and getting acquainted with people; shouldn't talk shop to people, but keep his eyes always open for the main chance; establish credit with the best merchants by running accounts and paying them promptly when presented; render every service as efficiently and agreeably as circumstances will permit.

Another correspondent thinks this dentist doesn't know how to increase his income to meet increased expenses. He would cut out the expense of an office assistant and do her work himself. He recommends better equipment for the office and the dentist that he may increase the quantity and quality of work done; use all known painless methods local as well as general anesthetics to popularize the practice; thinks the dentist is in a rut and should take a trip to the city to buy new appliances, not neglecting to inform the local newspaper of the trip; don't smoke, drink or loaf; but keep busy always with work, study or some form of outdoor exercise; increase fees steadily; keep out of all unknown money making schemes and make secure investments even if the interest is small. If discouraged and dissatisfied with the town move to some place where you and your family will be happy and well and start anew to build a reliable practice.

Another writes that most dentists make too many operations which they can't approve except as expedients and patients take for granted they are as good as can be done. Many patients woud have better services if they know they were available and needed. Many needed operations are overlooked and many patients are inclined to be satisfied with as slight an operation as possible, when more thorough services are clearly indicated and will receive grateful recognition in fees as well as appreciation if administered properly.

Another writer says it makes him indignant to think of a dentist with a wife and two children giving up "a modest little home on the edge of town" to kennelling like a lame hound in a flat back of a dental office. Out on the edge of town is just the place to live and bring up the children, just far enough away to give one needed exercise in walking to and from the office. As a remedy he would *buy* a small place on the edge of town, buy the wife and children some good clothes; take a lot of pride in and have a lot of fun and exercise in fixing up the home and place so that every body who passed would admire it and want to know who owned it. Then if necessary he would revise his fee bill to meet these expenses and take pains to educate the people to as good dentistry as their millinery at least.

We also print in this issue a communication from Dr. W. H. Trueman the veteran writer and practitioner which gives his views which are well worth consideration. Also several articles on the general subject which present the subject in ample detail for profitable consideration.

Some of the views reported above, we cordially endorse and know that a more careful consideration of the principles involved would make the practice of our profession a more successful and attractive business opportunity than many find it to be. We should not however lose sight of the fact that we are not to consider our calling a business enterprise, and think we are justified in using every means that the wisdom and cunning of the mere trader has found effective. Our relations with our patients are intimate and sacred, and we can not serve them on the same basis that we would use in trading horses. A proper dignity is considered an essential quality in a professional occupation. Necessarily it becomes so, not because we will it, but we are inevitably driven to it by our inherent sense of personal rights and social customs. If we undertake to do what all of these writers imply, if they do not actually so state it, we must take more than a traders interest in the services we render, and consequently the professional claim asserts itself eventually, even though we don't keep our eyes on the main chance." No dentist who puts much of his life energy into an exacting service for a patient can help acquiring a personal interest in the welfare and happiness of that patient, and he could not possibly degrade his conscience to the extent of serving such a one for mere gain. He will because of his good service elevate his ideals and dignify himself as well as his calling and may therefore be honestly entitled to a remunerative fee, which is usually recognized and willingly requited. The establishment of such a habit insures not only a grateful and sufficient clientele, but renders it unnecessary, if not distasteful, for one to resort to public display of any kind as a means of business promotion. One of the writers says the dentist in question is in a rut and should get out if he has to move to another town. This is probably good advice, but of what avail if he carries his rut with him, he will probably find plenty of other ruts like his in the new location and the last estate will, if anything, be worse than the first. Get out of the rut to be sure, but be sure that you mount a wheel with a tire that is broader than the one you have left behind or that has preceded you in your new field. Get a broader conception of your mission and learn to serve better than any predecessor and you will find people willing to compensate your efforts so that it will not be necessary for you to live meanly in the sight of any one. An honest motive and habit, and a determination to make one's life worth living in any calling will breed a determination that will provide the wherewithal for food and raiment. Prudent living and wisdom in saving are of course to be commended and should not be neglected by any thoughtful dentist, as the time always comes when these provisions are necessary; but it is a mistake to make money getting and serving the first and predominating characteristics, because it dwarfs one's soul and detracts from his usefulness and in the end gives little pleasure. Every dentist should carry enough life insurance to moderately at least protect his family and save enough to provide comfort in old age; but his life can be more profitably spent in providing health and happiness for his patients than in accumulating an excess of riches to damn his descendants. The trouble with most of us is that we are either so professional that we give our lives and services to unappreciating people who don't know enough of our sacrifice to properly compensate it, and we die paupers; or we get the money microbic infection which runs its course through ambition to selfishness and meanness. It is to be hoped that out of this discussion we shall come to a saner understanding of ourselves and of the nobility of the work to which we are called.

